# A Coevolutionary Residue Network at the Site of a Functionally Important Conformational Change in a Phosphohexomutase Enzyme Family

**DOI:** 10.1371/journal.pone.0038114

**Published:** 2012-06-07

**Authors:** Yingying Lee, Jacob Mick, Cristina Furdui, Lesa J. Beamer

**Affiliations:** 1 Department of Chemistry, University of Missouri, Columbia, Missouri, United States of America; 2 Department of Biochemistry, University of Missouri, Columbia, Missouri, United States of America; 3 Department of Internal Medicine, Wake Forest University Health Sciences Winston-Salem, North Carolina, United States of America; Monash University, Australia

## Abstract

Coevolution analyses identify residues that co-vary with each other during evolution, revealing sequence relationships unobservable from traditional multiple sequence alignments. Here we describe a coevolutionary analysis of phosphomannomutase/phosphoglucomutase (PMM/PGM), a widespread and diverse enzyme family involved in carbohydrate biosynthesis. Mutual information and graph theory were utilized to identify a network of highly connected residues with high significance. An examination of the most tightly connected regions of the coevolutionary network reveals that most of the involved residues are localized near an interdomain interface of this enzyme, known to be the site of a functionally important conformational change. The roles of four interface residues found in this network were examined via site-directed mutagenesis and kinetic characterization. For three of these residues, mutation to alanine reduces enzyme specificity to ∼10% or less of wild-type, while the other has ∼45% activity of wild-type enzyme. An additional mutant of an interface residue that is not densely connected in the coevolutionary network was also characterized, and shows no change in activity relative to wild-type enzyme. The results of these studies are interpreted in the context of structural and functional data on PMM/PGM. Together, they demonstrate that a network of coevolving residues links the highly conserved active site with the interdomain conformational change necessary for the multi-step catalytic reaction. This work adds to our understanding of the functional roles of coevolving residue networks, and has implications for the definition of catalytically important residues.

## Introduction

Recent developments in bioinformatics have provided new tools for understanding relationships between protein sequence, structure, and function. Analysis of amino acid coevolution using information theory is one approach that has proven useful for a deeper appreciation of sequence relationships within protein families, and as a basis for interpreting functional roles of the coevolving residues. Recent studies of coevolving residues have revealed roles in protein stability, enzyme catalysis, intermolecular interactions, and macromolecular recognition [1]–[7]. Methods such as coevolutionary analysis are increasingly necessary for deriving insights from the rapidly expanding quantities of sequence information, which far exceeds capacity for experimental investigation. As the methodologies for calculating coevolution continue to improve, this approach holds promise for providing insights as far-reaching and important as those routinely obtained from sequence conservation.

Herein we apply recent approaches in coevolution to study a diverse enzyme family known as phosphomannomutase/phosphoglucomutase (PMM/PGM) (EC 5.2.2.8). PMM/PGM proteins comprise a widespread enzyme family involved in prokaryotic carbohydrate biosynthesis. They represent one sub-group of the α-D-phosphohexomutase enzyme superfamily, according to their similar preference for glucose and mannose-based phosphosugar substrates [8]. The enzyme reaction entails an intramolecular phosphoryl transfer reaction, converting a 1-phosphosugar into the corresponding 6-phosphosugar. The reaction proceeds via a bisphosphorylated sugar intermediate, is highly reversible, and dependent on Mg^2+^. A well-studied PMM/PGM is the enzyme from the human pathogen *Pseudomonas aeruginosa* [9]–[18]. In this organism, PMM/PGM participates in the biosynthesis of several bacterial exoproducts involved in virulence of infections, including lipopolysaccharide, rhamnolipid, and alginate [19], [20]. In other bacteria, PMM/PGM proteins have varied biosynthetic roles and are also associated with virulence and resistance to antibiotics [21]–[28]. Thus these enzymes are of potential interest for the development of inhibitors with clinical utility against bacterial infections.

Structural and mechanistic studies of *P. aeruginosa* PMM/PGM have revealed key features of enzyme mechanism, including two distinct but overlapping binding modes for its 1- and 6-phosphosugar substrate and product [12]. Crystal structures of *P. aeruginosa* PMM/PGM have also shown that binding of ligand in the active site is accompanied by an interdomain conformational change of ∼10 degrees, via a hinge at the juncture of domains 3 and 4 of the protein [12]–[14]. This conformational change permits residues in all four domains of the enzyme to participate in ligand contacts, and positions the substrate appropriately for phosphoryl transfer. A unique feature of the PMM/PGM reaction is the required reorientation of the reaction intermediate, glucose 1,6-bisphosphate, which occurs in between the two phosphoryl transfer steps, with a necessary accompanying conformational change of the enzyme [11], [13]. The factors governing the conformational flexibility of the protein (e.g., sequence determinants, dynamic properties, etc.) remains a key area of interest with regard to the function of this enzyme, and others in the superfamily.

In the present study, a coevolution analysis was used to examine sequence relationships of enzymes in the PMM/PGM family. Mutual information analyses were used to identify coevolving residue pairs (i.e., residues that change together during evolution), and “cliques” were calculated using graph theory to find networks of coevolving residues. We identify a tightly linked network of coevolving residues, most of which localize to the interface between domain 4 and the rest of the protein, which is a well-characterized site of conformational variability in the enzyme [12], [18]. This result is in distinct contrast to the highly conserved residues in the protein family that cluster in the active site and tend to be directly involved in catalysis/ligand binding. Furthermore, we report the steady-state kinetic characterization of mutants of residues in the coevolving network, and find a reduction in enzyme specificity (*k_cat_*/*K_m_*) relative to wild-type (WT), ranging from 45 to less than 10%. Mutation of an interface residue that is not part of the coevolving cluster results in no change in specificity, despite making direct structural contacts with other residues in the network selected for mutation. Double mutants of several key residues in the network show additivity in their effects, suggesting that these residues are not significantly coupled energetically. This study sheds new light on the roles of coevolutionary networks in proteins and has implications for the definition of catalytic residues in enzymes, which, as shown here, can be distant from the active site.

## Results

### Coevolution Analysis by Mutual Information

A multiple sequence alignment (MSA) of 465 PMM/PGM sequences was assembled as described in Methods. The median overall sequence identity of the MSA is 43.8% for ∼400 ungapped positions. This MSA is highly robust for the mutual information (MI) analysis below, both in terms of finite sample size effects, which can occur in alignments with fewer than 150 sequences, and phylogenetic influence, which can arise when a number of closely related sequences are found in the MSA [29]. In our alignment, the large number of sequences helps reduce background MI from random pairings of residues that is problematic in small alignments, and the 90% sequence identity cutoff helps removes bias imposed by evolutionary history. It should be noted that in the case of the PMM/PGM family, the very high conservation of the active site residues (including those directly involved in catalysis/ligand binding) largely excludes these residues (∼30) from the coevolutionary analysis.

For the coevolutionary analysis, a Z-scored-product normalized mutual information (*ZNMI*) algorithm was employed [30]. When applied to sequence alignments, MI quantifies the reduction in sequence uncertainty for a pair of positions over what would be seen if the two positions were evolving independently. Several variations of MI were tested on our MSA, including *ZRes* [5], *ZNMI* [30], and *Zpx* [31]. The *ZNMI* approach was chosen as it gave results for our MSA that were better when considering both accuracy and reproducibility relative to the other MI methods (Fig. S1), using the definitions from [30]. Accuracy of the various methods was assessed by determining if residue pairs with high scores are, on average, close in tertiary structure, the most commonly used metric for evaluating coevolution analysis [29].

ZNMI was calculated between all ungapped positions in the PMM/PGM alignment as described in Methods. The residue couplings from *ZNMI* were then subjected to a resampling procedure to eliminate errors due to perturbations of the sequence alignment (e.g., inclusion or exclusion of certain sequences). For this purpose, ensembles of sub-alignments were utilized in a cross validation approach, as described in [30]. For the present study, only residues with 100% reproducibility as determined by the data resampling were considered for further analysis. This stringent cutoff greatly reduces the possibility of false positives from the MI analysis, and, given the large size of the protein, helps reduce the number of coevolving positions to a manageable number for analysis (see following paragraph). It does, however, have the potential disadvantage of missing significant couplings (false negatives), a limitation that must be considered in interpretation of results.

**Figure 1:**
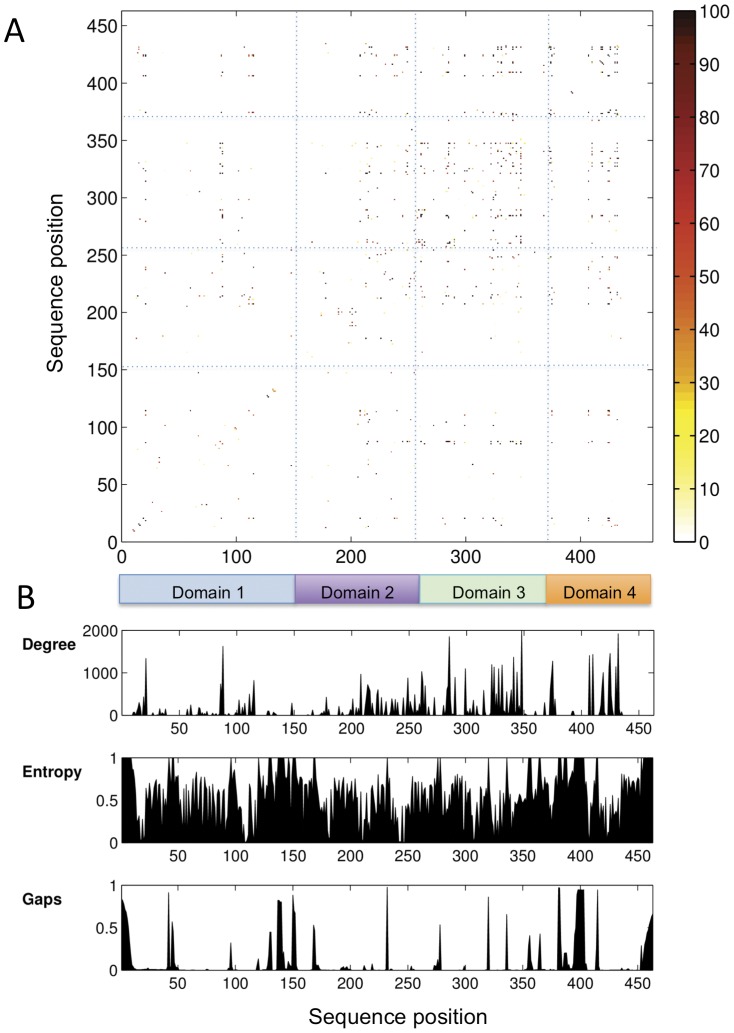
Results of the coevolutionary analysis. (A) A matrix of *rZNMI* scores by residue for the PMM/PGM MSA (lower right triangle). Range (0–100) indicates the reproducibility of the residue couplings from the data resampling (Methods). Blue dashed lines separate regions of the matrix according to the domain of the protein. (B) Plots showing the degree (summation of reproducible couplings), entropy and gaps for each column of the *rZNMI* matrix. Colored boxes at top indicate the four domains of PMM/PGM: domain 1 (residues 1–154), domain 2 (residues 154–256), domain 3 (residues 257–368), and domain 4 (residues 369–463); numbers according to the *P. aeruginosa* enzyme sequence.

A matrix of the resampled *ZNMI* (*rZNMI*) scores for all possible residue position pairs in the PMM/PGM MSA is given in Fig. 1A. Also, for comparison, Fig.1B shows per residue plots of the degree (summation of reproducible couplings), sequence entropy, and gaps for the MSA. The matrix in Fig. 1A is notably sparse due to application of the 100% reproducibility criterion. Despite this, the number of high-scoring residue pairs from the *rZNMI* analysis is still quite large (157 couplings over 74 sequence positions), and difficult to assess on an individual basis. In general, however, it can be seen that high-scoring couplings are scattered throughout the sequence, although with higher density in the C-terminal half of the protein. When considered according to specific domains, a propensity of high-scoring residue pairs occur within domain 3 (intradomain couplings) and between residues from domains 3 and 4 of the protein (interdomain couplings). (For reference, a structural overview of the domain architecture of PMM/PGM is given on Fig. S1). As expected, Fig. 1B shows that residues with high degrees of coupling do not coincide with regions of low sequence entropy (high conservation). It also shows that our MSA does not have extended continuous regions of high degree, which have been correlated with sequence misalignments [32].

### Identification of Residue Cliques

As noted above, the number of significant residue pair couplings from our *rZNMI* calculations is quite large (>150), despite our conservative data reprocessing approach. This highlights a drawback of coevolutionary analyses in all proteins, which is the difficulty of evaluating (experimentally or otherwise) the roles of the many pairwise residue couplings, particularly in the case of a large protein such as PMM/PGM. As an alternative, some recent studies have chosen to focus on coevolving residue “networks” [6], [33], [34], i.e. groups of coupled residues all of which co-vary with each other. Such an approach necessarily ignores individual high-scoring residue couplings that are independent from others, but has the advantage of reducing the coevolutionary couplings (and residues) under consideration to a number more conducive to detailed analysis. Moreover, networks of coupled residues have been found to highlight regions of functional importance [4], [6], [35].

To this end, we utilized a novel approach only recently applied to coevolutionary studies [36] to identify networks of coupled residues in PMM/PGM. Residue pair scores from the *rZNMI* calculations were assessed using cliques, a concept from graph theory for defining tightly connected regions of a network [37]. (The term network is used herein in its general sense of a group of related residues). Cliques have been used to characterize various types of networks, including both social and biological [38]–[40]. In the context of coevolution, a clique represents a set of residues wherein each residue covaries with all of the others. Residues in the same clique are referred to as neighbors. It is important to note that a given residue is not necessarily found exclusively in one clique, as a clique represents relationships between residues, not a list of independent residues.

Residue cliques for PMM/PGM were determined as in described in Methods. A total of 49 cliques containing two or more residues were identified, comprising a total of 66 unique residues out of the 463 possible sequence positions. Most of the cliques define connections between only two or three positions of PMM/PGM. However, some cliques are larger: three contain six residues and two contain five residues (Table 1). For further analysis, we chose to consider the union of all residues from these five largest cliques as they represent the densest, most interconnected region of the coevolutionary network. Previous studies have shown that coevolving positions in MSA that involve multiple other residues are frequently of functional importance [4].

**Table 1:** Residues of PMM/PGM in the five largest cliques (denoted A-E).

Resi. #	88	249	261	284	285	331	341	374	410	419	430	432
Type	*DEP*	*AFG*	*DSY*	*LPV*	*IKR*	*FY*	*DST*	*GP*	*RST*	*LN*	*RV*	*NRV*
A	X		X	X	X				X			
B		X				X		X		X	X	
C					X	X	X		X	X	X	
D					X	X	X			X	X	X
E						X	X	X		X	X	X
Domain	1	2	3	3	3	3	3	4	4	4	4	4

**Figure 2:**
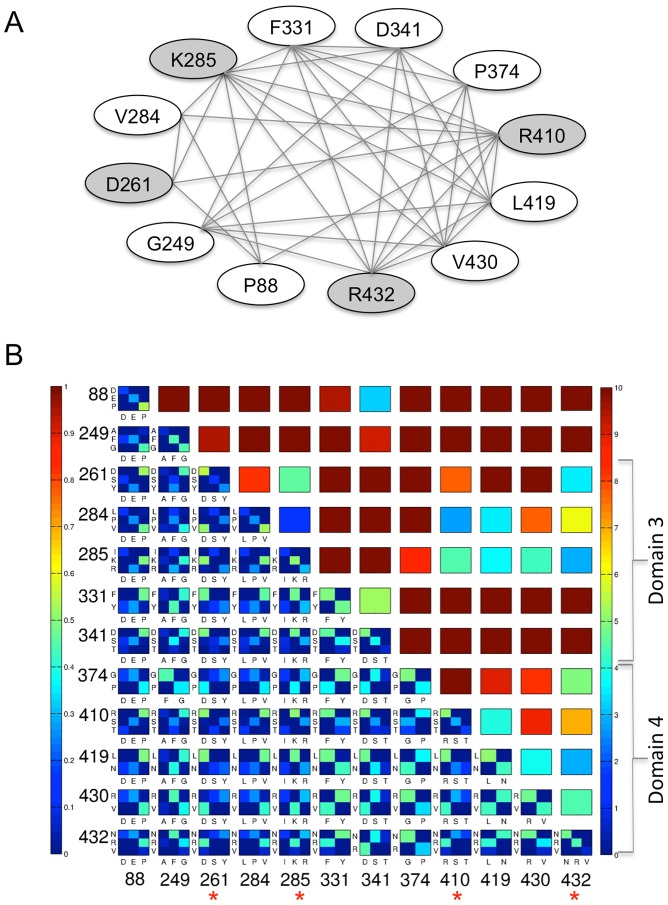
Results of the clique analysis. (A) Graph illustrating the connections between the 12 residues (ovals) in the top cliques of PMM/PGM (see text). Gray shading highlights residues characterized in mutagenesis studies. Lines connecting ovals indicate that two residues are neighbors (in the same clique). (B) **Upper right triangle:** A contact map showing the distance between the closest atoms for each pair of top clique residues, from 0 (blue) to 10 Å (red); see color bar. Residues belonging to domains 3 and 4 of the protein are highlighted by brackets on right. The physical proximity of top clique residues in the domain 4 interface can be easily visualized by the patches of blue/green. **Lower left triangle.** An array of bi-variate histograms showing the joint amino acid identities between the top clique residues. Axes for the array are residue numbers; each histogram is labeled with amino acid types along axes *i* and *j*. Blue indicates low joint residue identity (0.0); red indicates high (1.0). Joint identities that occur at a frequency of less than 5% were removed for simplicity. Each histogram is normalized to its sum, and since all residues shown are co-varying (i.e., not completely conserved), the maximal score (red) is not possible for any pair. Residues along the bottom axis highlighted by asterisks were subject to study by mutagenesis.

The union of the five largest cliques (hereafter called the “top cliques”) results in a set of just 12 residue positions of PMM/PGM, as many of the residues are found in more than one clique (Table 1). The network of relationships between the residues in the top cliques is illustrated in Fig. 2A, where each line connecting a pair of residues indicates that they are neighbors in a clique. Residue numbers on this figure and throughout manuscript refer to *P. aeruginosa* PMM/PGM. The connectedness of these residue positions varies considerably: some belong to only one clique (one set of neighbors), while other residues belong to multiple cliques and thus have many more neighbors. We note that each of the top cliques contains at least one residue from domain 3 of the protein and one from domain 4 (Table 1).

The clique analysis is a vast reduction of the coevolutionary data, and does not show, for example, the residue types and their frequencies at the co-varying positions. This information can be gained through examination of joint sequence identities, and this is shown for all 12 of the top clique residue positions on Fig. 2B (lower left hand triangle). This panel shows an array of bi-variate histograms for each possible pair of the top clique residues, even those that are not neighbors. Residue couplings are not explicit on this figure, which instead shows the initial data on joint sequence identity used in the MI calculations. However, by selecting neighboring positions (i.e., those connected by lines on Fig. 2A) the bivariate histograms can be used to find which residue types are found at each position, and their frequencies. Also shown in Fig. 2B (upper right hand triangle) is a contact map for the top clique residues, which highlights the physical proximity of many of top clique residues.

Some general observations can be made from Fig. 2B. For example, the top clique residues involve covariation between two or three different residue types. In some cases, the coevolving residue types that occur at a given position are quite similar (e.g., position 331: F/Y). Others coevolve as very different residue types, such as positions 374 (P/G) and 430 (R/V). One trend that might be expected from this data is that the physicochemical characteristics of the residue pair (e.g., apolar-apolar or charge-charge) would be conserved at co-varying positions, despite changes in residue identity. However, such patterns are not readily apparent for these residues. This may be due to the nature of the structural interactions made by the top clique residues, which are predominantly between atoms in the side chain of one residue and the backbone of the other (see following section).

### Structural Context of the Top Clique Residues

**Figure 3:**
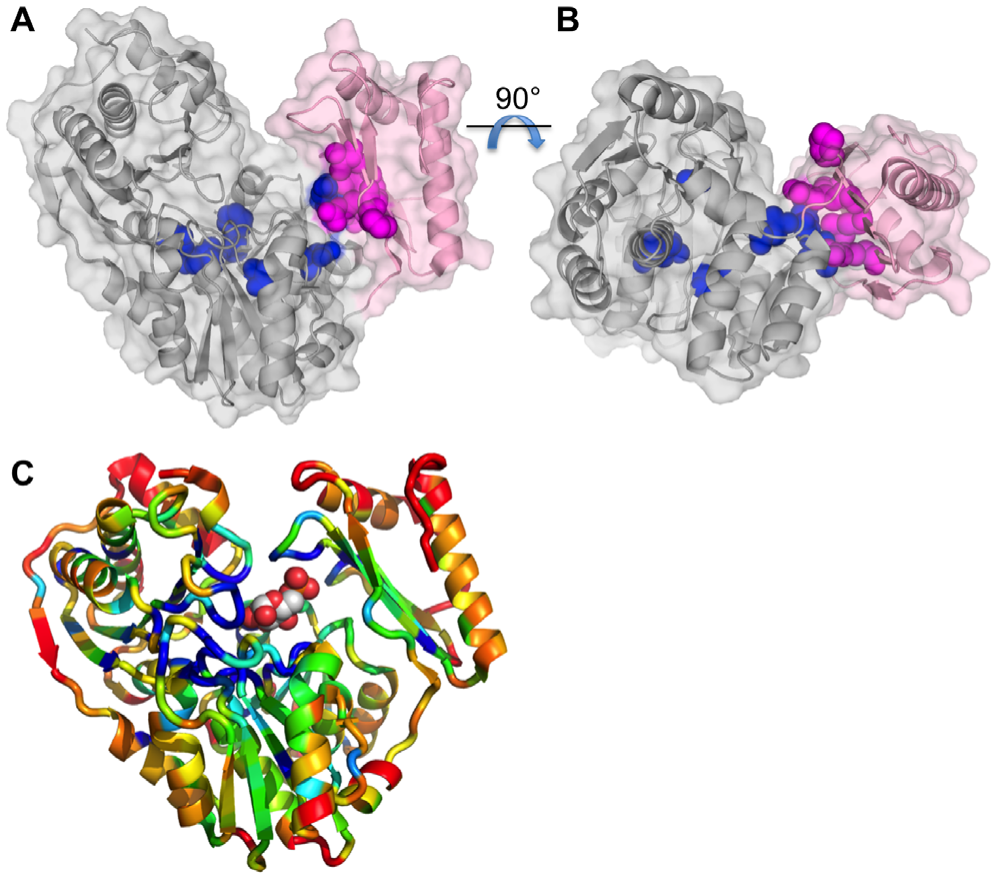
Structural context of the coevolutionary residue network. (A) Ribbon diagram of PMM/PGM from *P. aeruginosa* (PDB ID 1K2Y) with a semi-transparent surface. Domain 4 (residues 369–463) of the protein is shown in pink; the first three domains are gray. The 12 top clique residues identified by the coevolutionary analysis are highlighted as space filling models in blue (domains 1–3) and magenta (domain 4). (B) Same as panel A, but rotated by 90° for an alternate view of the domain 4 interface. Note that the top clique residues in the interface form a contiguous patch that spans the width of the interface. (C) Ribbon diagram of *P. aeruginosa* PMM/PGM (PDB ID 1P5G) colored according to sequence conservation. Glucose 1-phosphate is shown as a stick model with green carbons. Conservation is calculated according to an entropy score (see Methods and Fig. 1B) where blue color indicates high and red indicates low conservation. Residues that were more than 10% gapped were assigned a value of 1. Figure made with PYMOL [63].

The locations of the 12 top clique residues on the structure of *P. aeruginosa* PMM/PGM are shown in Fig. 3A. With only a few exceptions, it can be clearly seen that the top clique residues localize to a small region of the structure: the interface between domain 4 (pink) and the rest of the protein. The top clique residues fall on both sides of this interface, including residues from domains 3 (261, 284, 285) and 4 (374, 410, 419, 430, 432) of the protein. Indeed, these eight residues form an essentially contiguous residue patch (Fig. 2B), which spans the width (short dimension) of the domain interface (Fig. 3B). The remaining residues (those outside the domain 4 interface) are found in domains 1 (88), 2 (249), and elsewhere in domain 3 (331, 341), generally near the center of the molecule, but not within the active site cleft. With the exception of K285, which makes ligand contacts in certain enzyme-substrate complexes [12], none of the top clique residues were of previously implicated functional significance in PMM/PGM. However, the domain 4 interface is a known site of conformational change of the protein, as first observed in crystallographic studies of enzyme-substrate complexes (see Fig. S1 and Discussion for more detail) [12].

For comparison with the results of the coevolutionary analysis, Fig. 3C shows the structure of *P. aeruginosa* PMM/PGM colored according to sequence conservation in the MSA. On this figure, it is clear that the most highly conserved residues (blue) cluster in the center of the molecule, in or near the active site (see bound substrate for reference). In particular, clusters of conserved residues are found in active site loops in domains 1 and 2 of the protein. These loops include residues involved in the phosphoryl transfer reaction (S108) and coordination of the Mg^2+^ required for enzyme activity (residues 242–246) [12], [15]. Only a few highly conserved residues are found near the domain 4 interface. Hence, the structural location of highly conserved residues and coevolving residues in PMM/PGM are distinct, although if considered as a group, they would tend to form a contiguous patch in/near the active site, and extending along the domain 4 interface.

### Selection of Mutants and their Structural Interactions in the Domain Interface

To investigate the functional role of the coevolving network, a number of the top clique residues were selected for site-directed mutagenesis to alanine. Proline and glycine residues were excluded from consideration, due to possible negative effects on protein folding (P88, G249, P374). Of those remaining, residues were selected based on: i) their location at the interface between domain 4 and the rest of the protein; and, ii) a side chain involved in a hydrogen bond or ion pair interaction with another residue across the interface (e.g., one residue in domain 3 and the other in domain 4). Residues involved in bond interactions (rather than van der Waals contacts) were investigated, as they seemed more likely to show measureable effects on enzyme activity. This was a consideration due to previous studies that had suggested that mutation of a single residue within a coevolving network might have limited functional effects [1].

Residues and interactions in the domain 4 interface were identified from crystal structures of *P. aeruginosa* PMM/PGM [12], [15]. Three structures representing varying conformers of the protein were examined using the program DIMPLOT [41]. The residues and interactions in the interface depend on the conformer of the enzyme. 1K2Y is the most open of the PMM/PGM conformers, and has the smallest interface (673 Å^2^), while 1P5G represents the typical closed conformer observed in enzyme-ligand complexes, and has a larger interface (820 Å^2^). Overall, the total number of residues in the interface varies from 17 to 26 depending on conformer; a complete listing is shown on Table S1.

**Figure 4:**
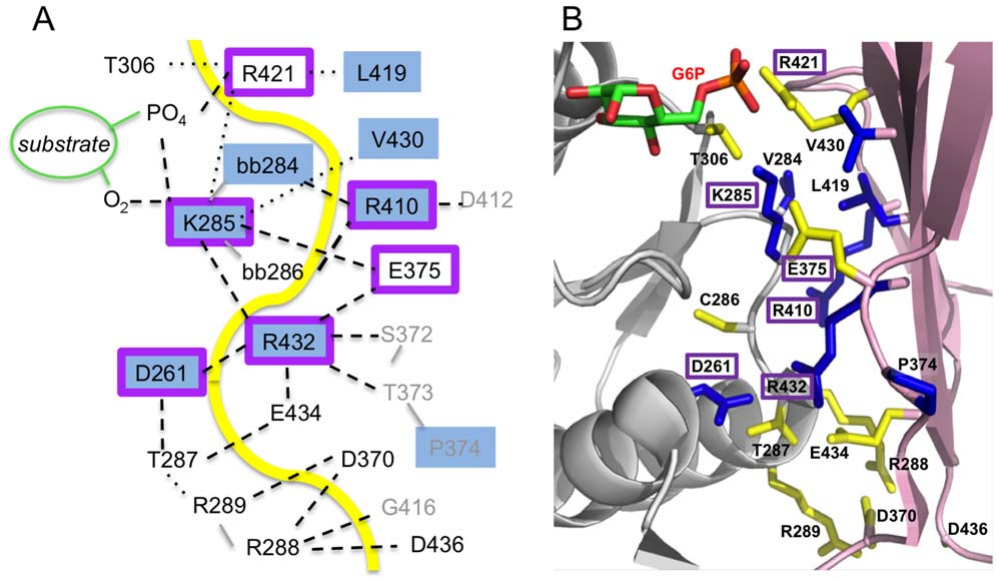
Structural networks in the domain 4 interface. (A) Schematic of the domain 4 interface of *P. aeruginosa* PMM/PGM, highlighting the residues involved and types of interactions. Hydrogen bonds are shown as dashed lines; van der Waals contacts as dotted lines. Yellow line approximates location of the inter-domain interface. Residues in the top cliques are highlighted with blue shading; residues selected for mutation (or previously mutated) are outlined in purple. Residues where contact involves backbone atoms are indicated with “bb” in the residue label; backbone connections between sequential residues are shown with a solid gray line. Non-interface residues are shown in gray font. Interactions represent a compilation of those seen in various crystal structures of PMM/PGM; not all interactions shown are found in each structure. See Table S2 for a full listing of interactions of clique residues. (B) A close-up of the domain 4 interface on the structure of PMM/PGM. Residues in the interface that are not top clique residues are shown in yellow; other colors as in panel A. Labels of residues selected for mutation are highlighted in purple boxes.

Four of the top clique residues meet the two criteria described above: D261, K285, R410, and R432. Their structural roles and interactions in the domain 4 interface are summarized in Fig. 4. This figure is a compilation of observed interactions as these vary depending on enzyme conformer and identity of bound ligand (see Table S2 for a full listing). D261 makes contacts with other residues in domain 3, and also across the domain interface with R432 in several of the enzyme-ligand complexes. K285 makes multiple contacts across the interface with residues in domain 4, including the backbone of R432. However, it is located on the periphery of the active site, and, as noted previously, also participates in ligand contacts. Hence, this residue acts as a bridge between the active site and the domain 4 interface, and is unique in this regard from the others. R410 contacts backbone atoms of residues 284 and 286, which flank K285. Most bond interactions between the top clique residues are between atoms in the side chain of one and the backbone of another. The only direct side chain-side chain interaction between two top clique residues is for D261 and R432.

In addition to the above, several other mutants were constructed and characterized, including E375A. Residue 375 is not a top clique residue, but is located in the domain 4 interface, and makes direct contacts with two of the top clique residues: K285 (inter-domain) and R432 (intra-domain). We note that E375 is identified in the clique analysis, but participates only in cliques of smaller size (two or three residues; data not shown) and has no neighbors in common with the top clique residues. Several double mutants were also constructed, using residues from among the single mutants of top clique residues that showed effects on kinetic parameters.

In this study, all residues selected for mutation were changed to alanine, in order to avoid introducing new structural interactions, and hopefully simplify interpretation of their effects. It is important to note that alanine does not occur naturally at any of the clique residue positions mutated herein (frequency in MSA is 0.0% for positions 261, 285, 410, and 432). For position 375, the frequency of alanine is also quite low (0.08%). Thus, as alanine is not frequently found at any of these positions in the MSA, each site should have a similar “baseline” for assessing affects due to the mutation. Other experimental approaches, such as mutating residues in a co-evolving pair to the alternative co-varying residues, were not pursued here, but may also be quite informative [31].

### Kinetic and Biochemical Characterization

The residues above were mutated to alanine, and mutant proteins were expressed and purified as described in Methods. Circular dichroism was used to verify correct folding and to determine apparent melting temperatures (T_m_) for each of the mutants (Table 2). Steady-state kinetic parameters for each protein were determined (see Methods), and the results summarized in Table 2. Relative to WT enzyme, each of the single mutants characterized shows a reduction in *k_cat_,* with the largest change being a 16-fold decrease for D261A, while E375A shows the smallest change with a modest 2-fold decrease in *k_cat_*. Effects on *K_m_* are less significant overall, with the largest change being a 2-fold increase for K285A. Comparison of *k_cat_/K_m_* for the single mutants relative to WT enzyme shows a range of effects from no difference (E375A) to more than 10% for D261, K285A and R432A. Overall, these results indicate significance for these residues in catalysis and substrate specificity, despite their location outside the active site of the enzyme. They are also consistent with our previous findings that conformational flexibility at the interface of domains 3 and 4 is critical to enzyme turnover [18]. The apparent T_m_ for the mutant proteins was quite similar to that of WT enzyme (Table 2), further supporting a role for these residues in catalysis, rather than protein stability.

**Table 2:** Steady state kinetic parameters for mutants of PMM/PGM in the conversion of glucose 1-phosphate to glucose 6-phosphate.

Protein	*k_cat_*	*K_m_* (µM)	*k_cat_*/*K_m_*	% rel. WT	CS	T_m_
	(s^−1^)	(µM)	(µM^−1^s^−1^)		(kcal)	(°C)
WT	6.96±0.19	28±3	0.25±0.03	-		62
D261A	0.43±0.02	54±7	0.008±0.001	3		58
K285A	1.682±0.009	66.7±1.1	0.0252±0.0004	10		68
E375A	3.02±0.09	11.5±1.5	0.26±0.03	100		58
R410A	2.07±0.03	18.5±1.3	0.112±0.008	45		60
R432A	0.96±0.02	40±3	0.024±0.002	10		59
K285A/R410A	0.89±0.02	56±4	0.016±0.001	6	−0.21±0.11	62
K285A/R432A	0.047±0.002	190±20	0.000247±0.000003	0.1	1.29±0.11	61
R410A/R432A	0.122±0.009	28±11	0.004±0.002	2	0.57±0.12	60

CS  =  coupling score.

In addition to the five single mutants, three double mutants were also characterized to assess energetic coupling between residues. By comparing the energetic perturbation of the double mutant with the sum of the perturbation caused by each mutation separately, the energetics of interaction between the two mutation sites can be determined. Double mutant cycles were constructed (see Methods) and changes in free energy of stabilization of the transition-state (ΔΔG^≠^_T_) calculated from *k_cat_/K_m_* using Eqs. 3 and 4 (Table 2). For each of the double mutants (K285A/R410A, K285A/R432A, and R410A/R432A), the observed effects on *k_cat_/K_m_* are similar to what would be expected from combining the single mutants (i.e., additive). Hence, the coupling scores are generally small (<1.5 kcal/mol), indicating a lack of significant energetic coupling between these residues [42]. This is true even when the two residues mutated were neighbors in a top clique (e.g., K285/R410 and K285/R432). Thus it seems that in the PMM/PGM interface the effects of changing two residues within a clique are independent, despite the physical proximity of the residues involved.

## Discussion

### Our Computational Approach

In this study we employ a combination of recent methodological advances in coevolutionary analysis via MI. These include normalization for sequence entropy, controlling for phyletic effects and variation due to inclusion/exclusion of different sequences in the MSA (100% reproducibility cutoff). All of these have been previously shown to improve the performance of MI calculations [29], [30], [43]. This conservative approach certainly results in the loss of some significant (e.g., “real”) couplings for PMM/PGM. We believe, however, that those remaining can be categorized as unambiguously coevolving, to the limit of understanding in the field. It is critical that conservative and reproducible results be obtained from coevolutionary analyses, in order for such studies to become widely adopted by experimentalists, and facilitate functional studies of the roles of these residues. Currently available methods for this, such as site-directed mutagenesis, are too laborious for efforts to be expended on flawed sequence alignments (i.e., too few sequences or evolutionarily biased) potentially resulting in non-robust MI couplings [29].

In the current study, we have chosen to utilize coevolutionary data to identify networks of coupled residues in PMM/PGM. A novel approach, the identification of residues cliques, was used to identify the maximum sub-graph of mutually coevolving residues within our protein family. Clique analysis is well known in graph theory and widely used in other fields, but its application herein to identify coupled residue networks in proteins is quite recent. This approach was used previously in a coevolutionary analysis of G protein-coupled receptors [36]. However, in that study, the maximal cliques for the graph were not solved explicitly, as was done in our analysis. Hence, we believe this study is the first full enumeration of coevolving residue cliques in a protein family. Clique analysis may prove generally useful as a straightforward and reproducible way to highlight residue networks that are otherwise not detectable within the massive amount of coevolutionary data. This is especially true for large proteins that have potentially (Nres^2^–Nres)/2 coupled residues. However, it should be noted that solution of the clique problem can also be computationally prohibitive, and may not be applicable to all systems.

### Coevolving Residue Networks and the Interdomain Rotation of PMM/PGM

Proteins in the PMM/PGM family are widespread across the kingdoms of life, being nearly ubiquitous in bacteria, and are also found in archaea and rarely in eukaryotes (see Protein Information Resource: http://pir.georgetown.edu/pirwww/, family PIRSF005849). Homologs from various organisms can exhibit quite low pairwise sequence identities (<30%). Nevertheless, despite the considerable overall sequence diversity of these enzymes, active site residues directly involved in bond breakage/formation and ligand contacts are extremely well conserved and allow sequences in the enzyme family to be easily identified [8]. However, residues important for other reasons (e.g., folding, stability, conformational flexibility) are not as easily deduced from sequence conservation, particularly in the case of large and diverse protein families such as PMM/PGM.

Our coevolution study of PMM/PGM reveals a contrast in functional roles between the highly conserved and coevolving residues in this enzyme family. Highly conserved residues are generally clustered within the active site of the protein and are frequently correlated with known roles in catalysis and ligand binding [12]. In contrast, the MI analysis shows a dense network of coevolving residues that localizes to the interface of domain 4, the primary site of conformational change when comparing apo-enzyme and enzyme-ligand complexes of PMM/PGM (Fig. S1) [12]–[14]. From structural studies, it is clear that this domain rotation must occur at several points in the multi-step reaction of PMM/PGM, including upon substrate binding, to permit reorientation of the reaction intermediate, and for product release [13]. Hence conformational change plays a critical role in the reaction mechanism of this enzyme.

Due to the functional importance of conformational change of PMM/PGM, it is not surprising that residues in the domain 4 interface are subject to evolutionary pressure. While residues in this interface are not as highly conserved as those that participate directly in bond breakage/formation or ligand contacts, the MI and clique analysis reveals that they are part of the most densely connected network of co-varying positions in the protein. Coevolution of these residues is consistent with their location in a domain-domain interface, where multiple compensatory sequence changes might be necessary to maintain the structural features that control the extent, variability, and/or fluctuation rates of the domain 4 movement. Thus it is particularly interesting that coevolving pairs are found to include residues that make direct interdomain interactions across the interface, as observed in the *P. aeruginosa* PMM/PGM crystal structures. Notably, three of the top clique residues (D261, K285, and R432) are involved in a 3-way structural network of hydrogen bonds and ion pairs that only occurs in the enzyme-ligand complexes, not the apo-protein (Table S2). Thus, despite the fact that this interaction is conformer dependent and therefore transient, it is nonetheless highlighted by coevolutionary patterns. Another striking feature of the clique analysis, which is also reflected in the protein structure, is that these residues form a network that connects the domain 4 interface with the active site of the enzyme. A key residue in this network is K285, which is involved in both interdomain and ligand interactions. Despite its participation in direct ligand contacts [12], the kinetic characteristics of the K285A mutant are quite similar to those of other clique residues in the interface, suggesting perhaps that its primary role is in the coevolving interdomain network, rather than in ligand binding.

### Roles of Coevolving Residues and Comparison with Other Studies

In the case of PMM/PGM, the coevolution analysis did not highlight residues directly involved in catalysis, but rather revealed a network of residues that participate in a more subtle functionality: conformational variability. This result differs somewhat from those highlighted by other studies. For example, in the KDO8P synthase enzyme family, a correlation between coevolving residues and protein stability was noted [1]. In a large analysis of Pfam families, an increased likelihood for coevolution was found between pairs of catalytic residues [5]. On the other hand, the location of coevolving residues at a site of conformational change in PMM/PGM is consistent with a recent study that showed clusters of highly coevolving residues in the flexible regions of proteins [35]. More generally, our results also agree with those of Gloor and coworkers, who have identified clusters of coevolving residues near molecular interfaces [4].

From studies of individual protein families, it seems that the conclusions derived from coevolution studies depend strongly on the inherent features of the protein family (fold, function, sequence diversity, etc.), the construction of the MSA [32], and possibly the goal of the researchers. Therefore, at least at present, it seems quite difficult to draw general conclusions regarding the functional roles of coevolving residues in proteins. Further work, including experimental studies, is necessary to help disentangle the phylogenetic, structural, functional, interactional, and stochastic components of coevolution [44].

### Impact of Interface Mutants on Enzyme Function

The detection of amino acids important to structure/function of proteins through site-directed mutagenesis is prohibitive, due to the labor involved and the complexity and multifactorial nature of residue interactions. Hence the use of computational tools to highlight key residue positions for experimental characterization is highly desirable. In this study, 12 residues of 463-residue PMM/PGM were found to participate in a tightly connected coevolutionary network at a site of conformational change. Mutants of four of the 12 top clique residues located in the domain 4 interface were experimentally characterized, and shown to have effects on enzyme specificity, ranging from 3 to 45% of WT enzyme. In general, these effects are modest, but still noteworthy, especially as most of the mutated residues are not located near the active site of the enzyme. Thus it appears that the clique analysis is generally successful at identifying a network of residues with functional import in PMM/PGM. However, as mentioned in Results, many other residues in the domain interface could also be relevant to function, but have not been highlighted by our approach due to our strict data reprocessing of the MSA and focus on residue cliques (rather than pairs of coevolving residues). Indeed, it has been recently estimated that the majority of residues in a protein may be of functional importance [45]. The experimental characterization herein supports the notion that coevolutionary analysis can be used to help identify functionally important residues of proteins, although interpretation of their biochemical role(s) it must still be assessed on a case-by-case basis.

Our structural analysis of the clique residues shows that many of these residues are part of a network of hydrogen bonding residues in the domain 4 interface. To probe the relationship between the coevolutionary network and the structural network, a mutant of E375 (not a top clique residue) was characterized. Interestingly, mutation of E375 had negligible effect on enzyme specificity, despite its direct bonding interactions with multiple top clique and other residues in/across the interface. While the sample size is too small to draw any firm conclusions, this result appears to suggest that the co-varying residue groups identified by the clique analysis segregate (at least to some extent) by their impact on function. This result is consistent with previous observations that networks with multiple co-varying positions are associated with functional importance, while those that vary as pairs tended to be residues involved in direct contacts [4].

Due to the localization of the top clique residues of PMM/PGM in the domain 4 interface, as opposed to the active site, it would appear that the effects of the mutations are not manifested via direct influence on bond breakage/formation or ligand binding, but rather by affecting the interdomain conformational change necessary for catalysis. Possible mechanisms for this include differences in the magnitude or rate of the interdomain conformational change, or small changes to the orientation of domain 4, such that it is no longer optimally positioned for binding/catalysis. In a previous study, mutations affecting the flexibility of the polypeptide backbone in the hinge region (at the junction of domain 3 and 4) of *P. aeruginosa* PMM/PGM were characterized, and their effects were found to be to be primarily entropic in nature, consistent with an increase in the conformational flexibility of the protein [18]. These mutants, which include residues R262, P368, and S369 (Table S1), show similar changes in steady-state kinetic parameters to the mutants of the top clique residues. Hence, it is possible that the changes due to the mutations in this study may derive from similar effects. We note that of these previously characterized hinge mutants, only one is found by the clique analysis: R262, which is involved in smaller cliques of size two. P368 and S369 are not found in any cliques, although P368 shows a moderate degree of coupling (Fig. 1B).

### Clique Residues Show Only Weak Energetic Coupling

Some early coevolutionary studies correlated coevolving residue positions in proteins with thermodynamic coupling [46]. To explore that possibility in PMM/PGM, three double mutants were characterized, selected from among the single mutants of the top clique residues, and two of which were between neighboring residues in a clique. Moreover, crystal structures of *P. aeruginosa* PMM/PGM show that these residues interact either directly with each other via hydrogen bonds or through contacts with sequentially adjacent residues (Fig. 4). Thus, they seemed reasonable candidates to examine for coupling, as it has been long established that residues physically close tend to exhibit thermodynamic coupling [47]. Despite this, double mutant cycles demonstrated generally weak or insignificant coupling between the residues examined in this study.

Although not conclusive due to the sparse sampling of residues selected for mutagenesis, our results appear generally consistent with recent analyses showing that co-varying residue positions are not good predictors of thermodynamic coupling [47], [48]. In the PMM/PGM family, it seems possible that the location of the coevolving residues at a site of conformational change, and hence variable residue interactions in the interface, might contribute to the observed lack of coupling. We note, however, that the contacts made between R410 and residues 284 and 286 are found in all conformers of PMM/PGM (Table S2), yet the K285A/R410A mutant still shows no energetic coupling. It may also be true that the current experimental approach (i.e., site directed mutagenesis) is less than optimal for verification of coupling within large residue networks. It is not clear *a priori* what types of functional effects or magnitude of coupling should be expected from changing one or two amino acids within a tightly connected network, such as the top clique residues described herein. Perhaps mutation of multiple residues would be necessary before significant coupling could be observed, but this approach could suffer from technical complications. Methods that can quickly examine relationships across multiple residues (e.g., hydrogen/deuterium exchange studies) are attractive alternatives, although they do not report directly on functional effects, such as changes in catalytic efficiency.

### Conclusions

In this study, we find that coevolutionary analysis highlights a subtle functionality of a protein family: conformational change. This result may be just a hint of the exciting new dimensions of knowledge that coevolutionary studies may glean from sequence comparisons. Such studies should prove especially informative and reliable in the case of large and diverse sequence families, where MI calculations are most robust. In addition, the clique analysis described herein can be easily applied to many other systems and provides a convenient method for identifying the largest networks of mutually coevolving residues. This provides a unique perspective on the massive quantity of data generated by coevolutionary analysis and may help focus the selection of residues for experimental characterization. It should be quite interesting to see what features are highlighted by residue cliques in other protein families, and to determine the relative roles and importance of coevolving residue networks versus residues pairs in protein function.

The coevolutionary residue network in PMM/PGM highlighted residues outside of the active site of the enzyme, and a role for these residues in catalytic efficiency was then demonstrated by kinetic characterization. This result is consistent with a number of other recent experimental studies showing the importance of residues outside the active site in catalysis [49]–[54], suggesting that an expansion of the definition of “catalytic residues” may be necessary [55]. More generally, it appears that coevolving residues and networks of residues may help explain the large size of enzymes, which typically comprise many more residues than those directly involved in ligand interactions, and the making or breaking of chemical bonds [56].

## Methods

An overview of computational methods in the following sections is presented as a flow chart in Fig. S2. Unless otherwise specified, all calculations were performed with MATLAB.

### Sequence Processing

Sequences of PMM/PGM proteins were downloaded from the PIR database (http://pir.georgetown.edu/pirwww/dbinfo/pirsf.shtml). Sequences were retrieved by searching the protein name field for phosphomannomutase or phosphoglucosamine mutase. (Both protein names were used in searches to maximize the number of retrieved sequences, as these two closely related families are frequently mis-annotated in database entries due to their sequence similarity). A multiple sequence alignment was constructed with MUSCLE v3.8.31 [57]. Sequences with less than 400 amino acids or greater than 500 amino acids were removed. A 90% sequence identity cutoff was imposed, resulting in 1189 sequences.

As noted in the Introduction, PMM/PGM proteins are one sub-group of the large α-D-phosphohexomutase superfamily, all members of which share highly conserved active site residues, and thus are easily identified from amino acid sequence. The PMM/PGM proteins are easily separated from two sub-groups in the superfamily, the phosphoglucomutases and phosphoacetylglucosamine mutases, due to differences in sequence length (PMM/PGMs are typically ∼450 residues in length, while the others are ∼550). However, the PMM/PGM proteins are of similar length to another sub-group in the superfamily, the phosphoglucosamine mutases (PNGMs), which have a different specificity for the sugar portion of the substrate. Hence, sequences of both PMM/PGM and PNGM proteins were found in the initial alignment.

As the presence of paralogs in the MSA could reduce utility of *ZNMI* for mutagenesis of residues in PMM/PGM [32] (the two proteins could be under different evolutionary constraints), we used a novel spectral clustering approach to separate these two closely related enzyme sub-groups. This method avoids pairwise metrics and allows for analysis of global sequence relationships. Briefly, the method of Paccanaro, et al., [58] was employed, but using a simpler metric for sequence identity that does not take into account physico-chemical similarity. In addition, oscillations in cluster variance were addressed by normalizing the distance metric by the product of the mean of each residue’s n/2 nearest neighbors, where n is the number of sequences present [59]. Percent identity was defined to be the number of identical amino acids divided by the total number of amino acids at non-gapped positions. All elements of this matrix were subtracted from one to give distances ranging from 0 to 1, where high values indicate the most distant sequences.

Spectral clustering was then performed on the initial alignment above (all sequences and positions) as in [59]. This separated the sequences in the alignment into two groups (Fig. S3): 724 PNGMs, which were discarded for this study; and 465 PMM/PGMs, which were utilized for the coevolutionary analysis. The PNGM cluster was identified based on it containing multiple annotations for the *GlmM* gene product (phosphoglucosamine mutase) in biochemically characterized members of this protein family. The remaining sequences were assigned to the PMM/PGM sub-group. Prior to the *ZNMI* calculations, the MSA was truncated to include only sequence positions of *P. aeruginosa* PMM/PGM. Plots of sequence entropy and gapped residues for the final MSA are shown in Fig. S4. The MSA used in these calculations is available as File S1.

### Mutual Information

Information entropy (*H*) is a measure of uncertainty associated with a random variable [37]. Eq. 1 was used to calculate entropy for each column in the MSA that was <10% gapped. The entropy of a column c in the alignment was determined as shown in the following equation:
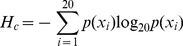
(1)Here, *p*(*x_i_*) is the observed frequency of amino acid *i* occurring at a site. All values were calculated using a log_20_ scale so that the range of position entropy scores was 0–1 (where c the column in the alignment). These extreme values occur when one amino acid is completely conserved or when each of the 20 amino acids occurs in a column with the same frequency. The joint entropy *H*_cd_, where c and d are columns in the MSA, was calculated by the same method using the frequencies of occurrence of each combination of residues at positions c and d. If two columns in the MSA are independent of each other, then the joint entropy will equal the sum of the individual entropies, *H*_c_+*H*_d_.

Mutual information was calculated as follows: MI_cd_ = *H*_c_+*H*_d_−*H*_cd_ [4]. The MI scores ranged from 0 to the minimum of *H*_c_ or *H*_d_. Normalized MI (*NMI*) was calculated by dividing MI by the joint entropy of the positions, *H*_cd_, to eliminate the influence of entropy on MI [29]. Values of the *NMI* range from 0 to 1.

Assuming a normal distribution for each residue pair *i, j* of the *NMI* matrix, a bivariate Z-score was assigned as follows. Given the NMI distribution N, the mean (µ *_i,j_*) and variance at residues (δ^2^ *_i,j_*), then
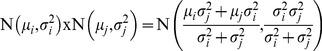
(2)For comparison of MI algorithms, *Zpx* and *ZRes* were implemented according to [5], [31].

### Data Resampling

To assess the reproducibility of the *ZNMI* couplings, an ensemble of partition alignments was created and used in a cross-validation approach, as originally described in [30]. For our case, a subset of 300 sequences from the PMM/PGM alignment was selected at random and divided in half (each set of 150 is referred to as a “split”), and scored by *ZNMI*. From each split, we extracted the top 462 residue pair couplings from the total of (463^2^−463)/2 or 106,953. This cutoff produces an average Z-score of 3.5 over all couplings in a split, similar to the Z-score of ∼4 found to be reliable for identifying coevolving positions in a different coevolutionary metric derived from MI [29]. A consensus of the top scoring couplings present in both splits was recorded, and the ratio of consensus couplings to 462 was used to determine reproducibility. This process (starting with random selection of another 300 sequences) was repeated a total of 100 times, and a compilation of the couplings that appeared in the consensus of *all* splits was extracted (100% reproducible). This approach emphasizes the couplings that are reproducibly significant for the entire network, eliminating all but the very highest ranked couplings.

### Identification of Residue Cliques

To identify maximal networks of residues with high coupling scores from the resampled *ZNMI* data, the “clique” concept from graph theory was utilized. The clique problem, which refers to finding a completely connected set of elements in a graph, was solved for the 100% reproducible *ZNMI* couplings derived from the PMM/PGM MSA. Maximal cliques were found using the NetworkX module in Python [60]. Due to the sparseness of the resampled *ZNMI* matrix, the computational time necessary to solve the clique problem was not prohibitive (161 sec on a 2.26 GHz quad core).

### Materials

*Leuconostoc mesenteroides* glucose 6-phosphate dehydrogenase and α-D glucose 1-phosphate, glucose 1,6-bisphosphate were obtained from Sigma-Aldrich. Anion exchange column chromatography was utilized to remove α-D glucose 1,6-diphosphate contaminants in α-D-glucose 1-phosphate [61].

### Site-directed Mutagenesis, Protein Expression and Purification

PMM/PGM mutants were constructed using the QuikChange kit (Stratagene) and verified by automated DNA sequencing. For expression of His-tagged wild type PMM/PGM and mutants, *Escherichia coli* BL21(DE3) cells were transformed with corresponding plasmids in pET14b vector. Initial cultures were grown at 37°C in LB media, supplemented with 0.1 mg/mL ampicillin, to an OD_600_ reading of 0.8 to 1.0. Prior to induction with isopropyl β-D-1-thiogalactopyranoside (final concentration 0.4 mM), cultures were cooled at 4°C for at least 30 minutes. Cells were induced for 12–16 hours at 19°C, the cell pellet collected by centrifugation, and stored at −80°C until further use.

For purification, cell pellets were resuspended in Buffer A (20 mM sodium phosphate, 0.3 M NaCl, pH 7.8) containing 14.4 mM β-mercaptoethanol, 0.5 mM phenylmethylsulfonyl fluoride, 0.5 mM *N*_α_-Tosyl-L-lysine chloromethyl ketone hydrochloride, 2 mM CaCl_2,_ 2 mM MgSO_4_, and 10 µg/mL DNase. Cell lysis was performed with a French press, and the soluble fraction containing PMM/PGM was obtained through centrifugation. Protamine sulfate was added at 5 mg/g of cell pellet over 15 minutes, stirred for 30 minutes, and centrifuged. The supernatant was mixed with Ni^2+^ affinity resin (His-Select, Sigma), which had been previously equilibrated in Buffer A, and incubated for 30 minutes on a two-way orbital rocker. The mixture was transferred into a gravity-packed column and washed with Buffer A containing 5 mM, and then 10 mM imidazole, pH 7.8. Protein was eluted using Buffer A supplemented with 250 mM imidazole, pH 7.8. Purified proteins were dialyzed by a slow NaCl gradient into 50 mM MOPS, 1 mM MgCl_2,_ 0.1 mM EDTA, pH 7.4, and then into the final buffer (50 mM MOPS, 1 mM MgCl_2_, pH 7.4).

### Steady-state Kinetics Studies

Enzymatic activities for wild type PMM/PGM and mutants were quantified by measuring the phosphoglucomutase activity in the direction of glucose 6-phosphate formation, using a coupled assay with glucose-6-phosphate dehydrogenase as previously described [18], with minor modifications. Enzyme concentration varied from 0.1 to 2.3 µM, depending on activity. The substrate (α-D glucose 1-phosphate) concentration was varied from 10–800 µM, depending on the amount of enzyme used. The activator glucose 1,6-bisphosphate was present at 1.0 µM, which was sufficient to relieve substrate inhibition in all proteins [10], except for the E375A mutant where it was increased 1.5 µM. Data were fitted to the Michaelis-Menten equation using SigmaPlot v12.0 ©. A control assay using WT enzyme was performed in parallel with the characterization of each mutant, to ensure that differences in the kinetic parameters observed for the mutant proteins were not due to changes in experimental conditions. All assays were performed in duplicate or triplicate.

### Double Mutant Cycles

Double mutant cycles were constructed to investigate the energetic coupling between two positions (X and Y) in PMM/PGM. Changes in transition-state stabilization energy (ΔΔG^≠^_T_) of single and double mutants relative to the WT enzyme were calculated by Eq. 3, where *R* is the gas constant and *T* is the absolute temperature in Kelvin. Eq. 4 was applied for the calculation of coupling energy (ΔG_I_) [62].
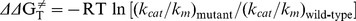
(3)

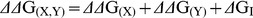
(4)


### Circular Dichroism

Protein samples (8–17 µM) in 10 mM MOPS, pH 7.4, were analyzed at 25°C in a 0.1 cm quartz cuvette with an Aviv 62DS spectrometer. Background subtraction was performed using buffer dialysate as the reference. Data were collected at 1 nm intervals from 190 to 250 nm and signal averaged for 30 seconds. For thermal denaturation, samples were heated from 25°C to 75°C while monitoring ellipticity θ at 222 nm. As thermal denaturation of PMM/PGM is not reversible due to precipitation of the protein at high temperature, the apparent *T_m_* reports on both thermal stability and kinetics of unfolding.

## Supporting Information

Figure S1 **Plots comparing the accuracy and reproducibility obtained with various MI algorithms on our MSA.** Algorithms were implemented as described in the following references: *ZRes* [5], *ZNMI* [30], and *Zpx* [31]. Reproducibility and accuracy are defined as in [30], and calculated using the top-scoring residue couplings.
(TIF)

Figure S2 **Superposition of three conformers of PMM/PGM, showing the variable orientation of domain 4.** Structures shown are 1K2Y (apo S108A mutant), 1K35 (WT apo-enzyme), and 1P5G (enzyme-substrate complex). Protein is colored by domain: domain 1 (residues 1–154) is yellow, domain 2 (residues 154–256) is green, domain 3 (residues 257–368) is blue, and domain 4 (residues 369–463) is pink.
(TIFF)

Figure S3 **Flow chart of computational steps described in Methods.**
(TIFF)

Figure S4 **Results of spectral clustering used to separate the PMM/PGM proteins from the closely related PNGMs.** Figure shows calculations from final version of MSA (truncated to ungapped residue positions of *P. aeruginosa* PMM/PGM). Numbering on axes refers to an arbitrary sequence index assigned to each protein. (A) Matrix showing the % sequence identity between each pair of sequences in the MSA, and permuted according to cluster indicator (each sequence was assigned to either the PMM/PGM or PNGM cluster). (B) Plot of sequence identity for the sequences in panel A relative to *P. aeruginosa* PMM/PGM. Overall, sequences in the PMM/PGM cluster are more similar to the *P. aeruginosa* protein, than those in the PNGM cluster, although there is a fair amount of sequence diversity within the PMM/PGM cluster. Sequences within the PNGM family are also somewhat diverse, but are all similarly equidistant from *P. aeruginosa* PMM/PGM, and hence show much less scatter.
(TIFF)

Table S1 **Residues in the interface with domain 4 of** ***P. aeruginosa*** **PMM/PGM.**
(PDF)

Table S2 **A summary of bond interactions between the top clique residues selected for mutagenesis and other residues in various crystal structures of PMM/PGM.**
(PDF)

File S1 Sequences in fasta format of the PMM/PGM proteins from the MSA, after truncation of the sequences to the structural template 1P5D (see Methods), as used for the *rZNMI* calculations.
(FA) Click here for additional data file.
