# Supplementary figures and images for: A Coevolutionary Residue Network at the Site of a Functionally Important Conformational Change in a Phosphohexomutase Enzyme Family
Source: PLoS One. 2012 Jun 7;7(6):e38114. doi: 10.1371/journal.pone.0038114 (PMC3369874; doi:10.1371/journal.pone.0038114)

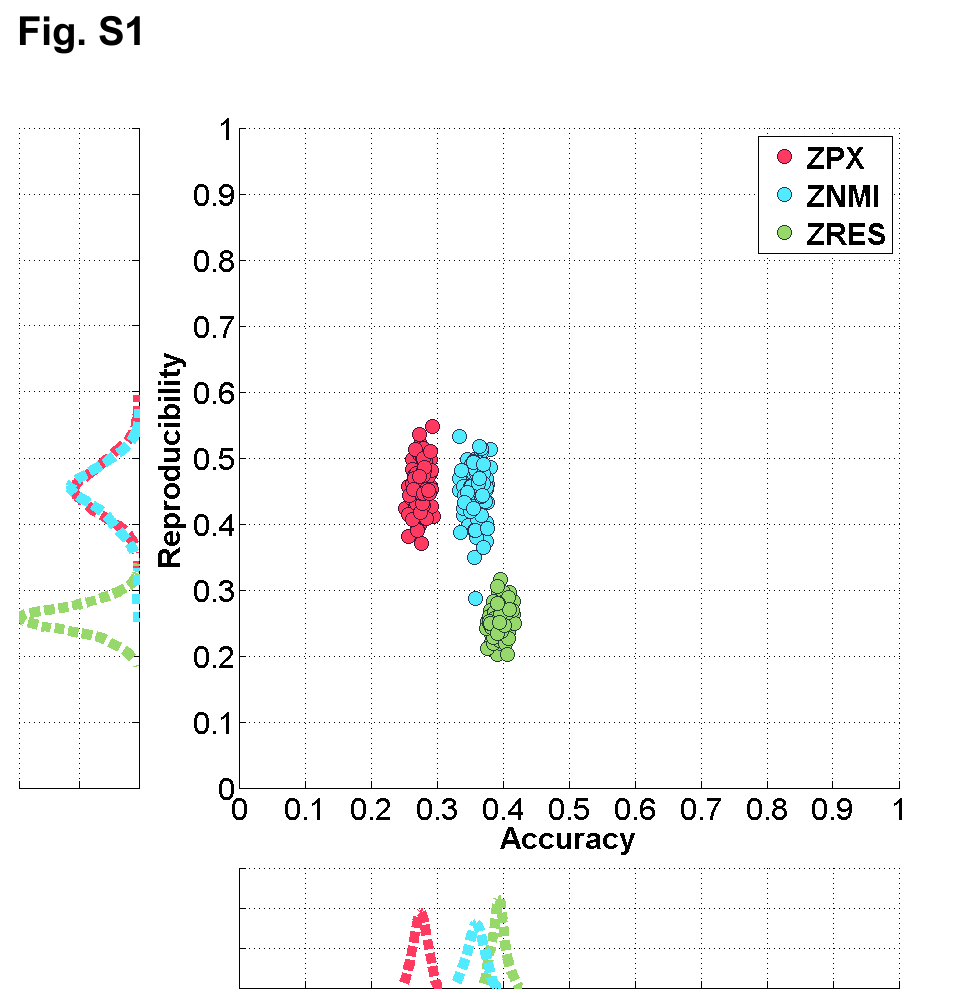

Supplement: Figure S1 — Plots comparing the accuracy and reproducibility obtained with various MI algorithms on our MSA. Algorithms were implemented as described in the following references: ZRes [5], ZNMI [30], and Zpx [31]. Reproducibility and accuracy are defined as in [30], and calculated using the top-scoring residue couplings. (TIF) [file pone.0038114.s001.tif]

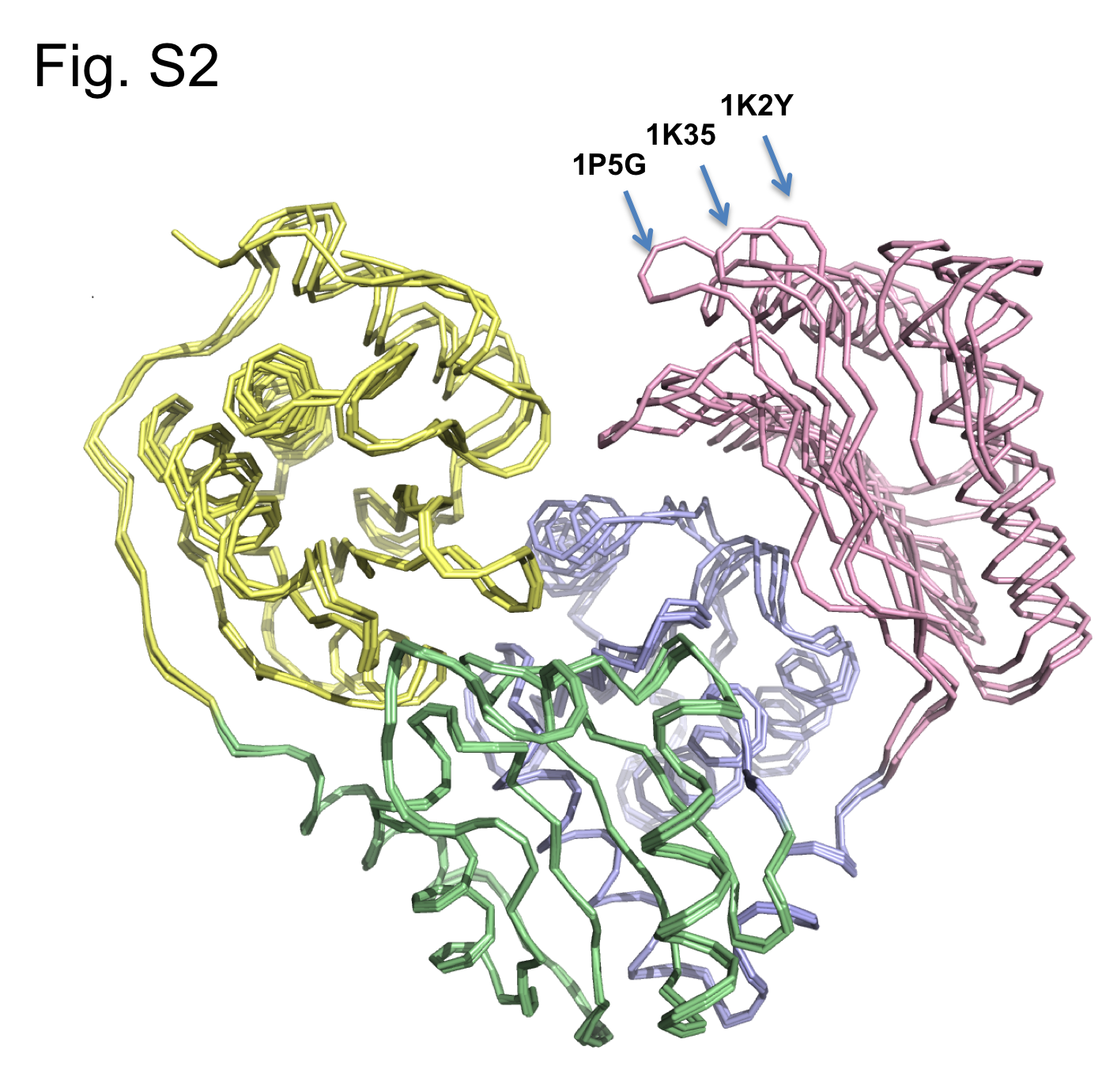

Supplement: Figure S2 — Superposition of three conformers of PMM/PGM, showing the variable orientation of domain 4. Structures shown are 1K2Y (apo S108A mutant), 1K35 (WT apo-enzyme), and 1P5G (enzyme-substrate complex). Protein is colored by domain: domain 1 (residues 1–154) is yellow, domain 2 (residues 154–256) is green, domain 3 (residues 257–368) is blue, and domain 4 (residues 369–463) is pink. (TIFF) [file pone.0038114.s002.tiff]

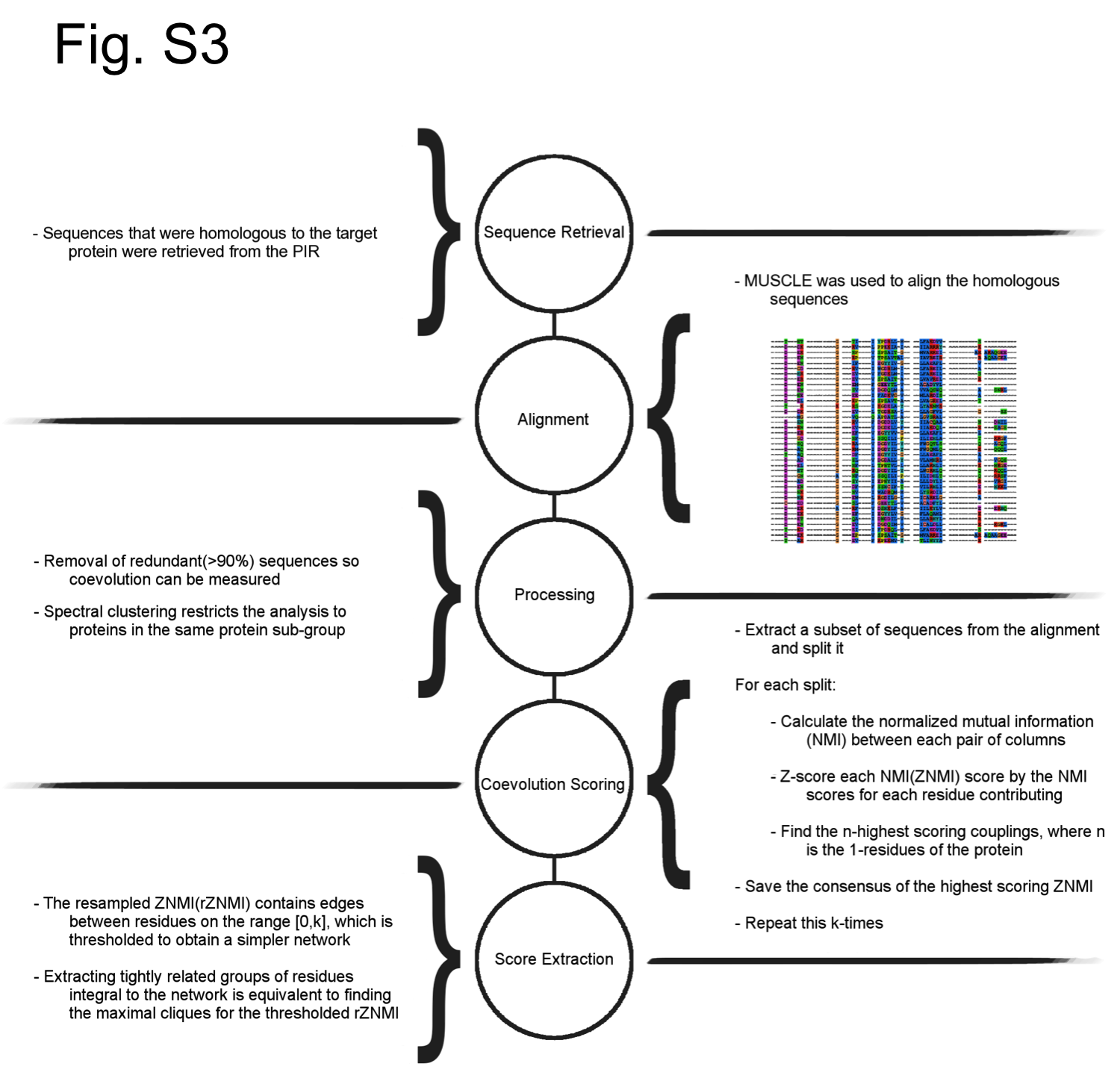

Supplement: Figure S3 — Flow chart of computational steps described in Methods. (TIFF) [file pone.0038114.s003.tiff]

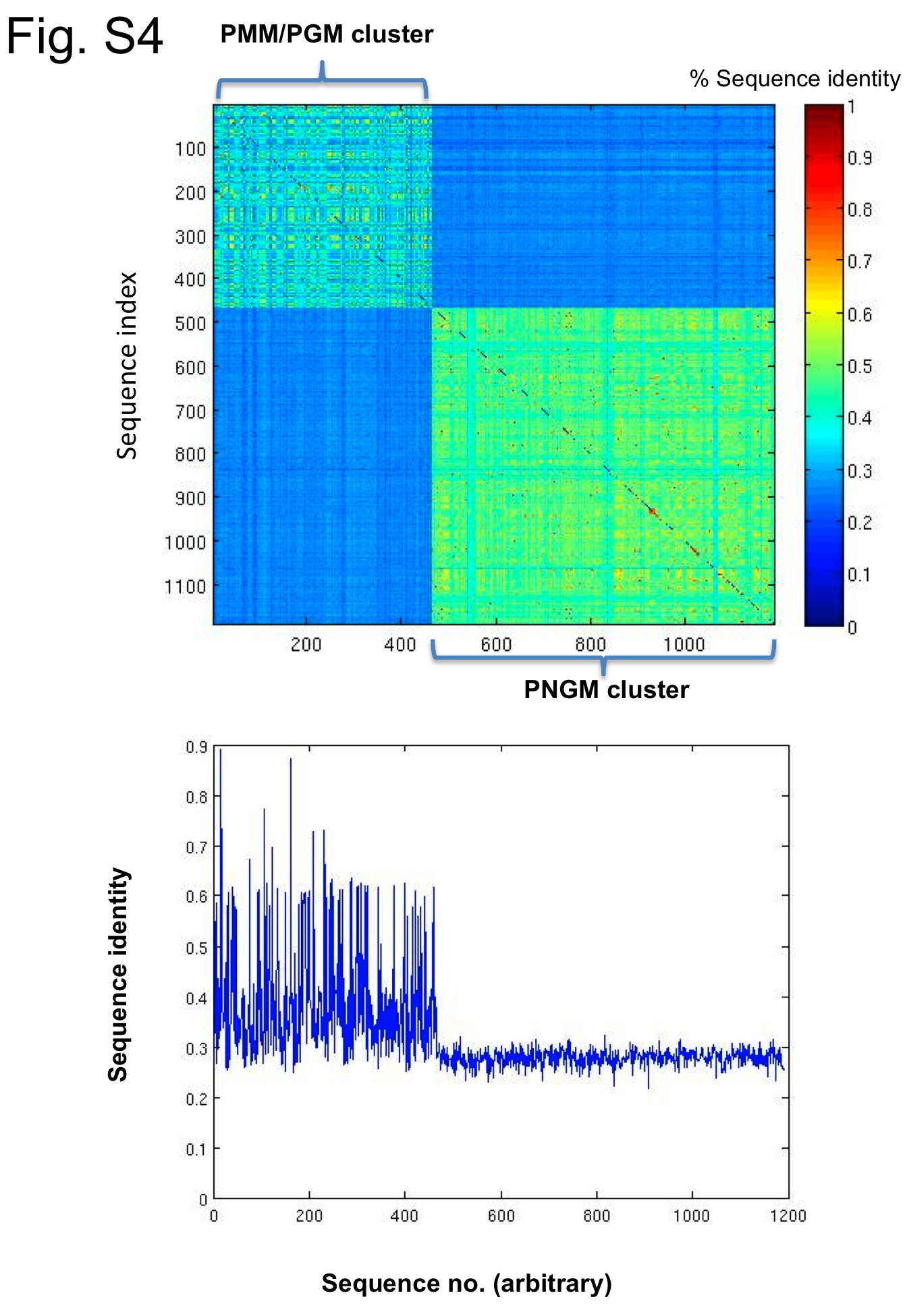

Supplement: Figure S4 — Results of spectral clustering used to separate the PMM/PGM proteins from the closely related PNGMs. Figure shows calculations from final version of MSA (truncated to ungapped residue positions of P. aeruginosa PMM/PGM). Numbering on axes refers to an arbitrary sequence index assigned to each protein. (A) Matrix showing the % sequence identity between each pair of sequences in the MSA, and permuted according to cluster indicator (each sequence was assigned to either the PMM/PGM or PNGM cluster). (B) Plot of sequence identity for the sequences in panel A relative to P. aeruginosa PMM/PGM. Overall, sequences in the PMM/PGM cluster are more similar to the P. aeruginosa protein, than those in the PNGM cluster, although there is a fair amount of sequence diversity within the PMM/PGM cluster. Sequences within the PNGM family are also somewhat diverse, but are all similarly equidistant from P. aeruginosa PMM/PGM, and hence show much less scatter. (TIFF) [file pone.0038114.s004.tiff]
