# Supplementary material for: A Coevolutionary Residue Network at the Site of a Functionally Important Conformational Change in a Phosphohexomutase Enzyme Family
Source: PLoS One. 2012 Jun 7;7(6):e38114. doi: 10.1371/journal.pone.0038114 (PMC3369874; doi:10.1371/journal.pone.0038114)
Supplement: Table S1 — Residues in the interface with domain 4 of P. aeruginosa PMM/PGM. (PDF) [file pone.0038114.s005.pdf]

**Table S1. Residues in the interface with domain 4 of *P. aeruginosa* PMM/PGM.**

| Crystal Structure | | | Site of Mutation |
|---|---|---|---|
| 1K35 | 1K2Y | 1P5G | |
| | | **D261** | * |
| | | **R262** | ‡ |
| | **V284** | **V284** | |
| **K285** | **K285** | **K285** | * |
| **C286** | **C286** | **C286** | |
| T287 | T287 | T287 | |
| R288 | R288 | R288 | |
| R289 | R289 | R289 | |
| | **M303** | **M303** | |
| **W304** | **W304** | **W304** | |
| | | **K305** | |
| **T306** | **T306** | **T306** | |
| | | **M326** | |
| **P368** | **P368** | **P368** | ‡ |
| S369 | S369 | S369 | ‡ |
| D370 | D370 | D370 | |
| E375 | E375 | E375 | * |
| **L406** | **L406** | **L406** | |
| D407 | D407 | D407 | |
| **R410** | **R410** | **R410** | * |
| W417 | W417 | W417 | * |
| **L419** | **L419** | **L419** | |
| | | R421 | ‡ |
| | **V430** | **V430** | |
| | | **R432** | * |
| E434 | E434 | E434 | |

Residues in bold were identified in top clique network. * indicates mutant characterized in this study; ‡ indicates mutant made previously (ref). Interface residues were identified using DIMPLOT [40]. The three crystal structures (1K2Y, 1K35, and 1P5G) represent varying conformers of the enzyme (see text).
