# Supplementary material for: A Coevolutionary Residue Network at the Site of a Functionally Important Conformational Change in a Phosphohexomutase Enzyme Family
Source: PLoS One. 2012 Jun 7;7(6):e38114. doi: 10.1371/journal.pone.0038114 (PMC3369874; doi:10.1371/journal.pone.0038114)
Supplement: Table S2 — A summary of bond interactions between the top clique residues selected for mutagenesis and other residues in various crystal structures of PMM/PGM. (PDF) [file pone.0038114.s006.pdf]

Table S2. A summary of bond interactions between the top clique residues selected for mutagenesis and other residues in various crystal structures of PMM/PGM.

Clique residue	1K2Y	1K35	1P5D (G1P)	1P5G (G6P)	1PCM (M6P)	1PCJ (M1P)
D261- sc	-	-	-	R432- sc	R432- sc	R432- sc
D261- bb	-	-	-	-	-	-
K285- sc	E375- sc	E375- sc	-	E375- sc	E375- sc	-
K285- bb	-	-	R432- sc	R432- sc	R432- sc	R432- sc
R410- sc	V284- bb	V284- bb	V284- bb	V284- bb	V284- bb	V284- bb
	C286- bb	C286- bb	C286- bb	C286- bb	C286- bb	C286- bb
R410- bb	-	-	-	-	-	-
R432- sc	-	-	K285- bb	K285- bb	K285- bb	K285- bb
	-	-	-	D261- sc	D261- sc	D261- sc
R432- bb	-	-	-	-	-	-

sc = side chain; bb = backbone atom; dash indicates no interaction. Substrates in enzyme-ligand complexes are indicated in parentheses (G1P: glucose 1-phosphate, G6P: glucose 6-phosphate, M1P: mannose 1-phosphate, M6P: mannose 6-phosphate). Contacts with top clique D261 occur only with R432 and are listed under interacting partners. Interactions calculated with DIMPLOT [40].
